# A Comparison of Training, Injury, Illness, Sleep, Wellbeing and Stress Between Developing Elite and Recreational Athletes

**DOI:** 10.1002/ejsc.70093

**Published:** 2025-11-23

**Authors:** Megan Lowery, Samuel J. Oliver, Ross Roberts, Clare Barwood, Emily Dunn, Eleanor Langham‐Walsh, Ben Holliss, Lizzie Wraith, Tim Woodman, Gavin Lawrence, Victoria M. Gottwald, James Hardy

**Affiliations:** ^1^ Institute for the Psychology of Elite Performance College of Medicine and Health Bangor University Bangor UK; ^2^ Institute for Applied Human Physiology College of Medicine and Health Bangor University Bangor UK; ^3^ UK Sports Institute London UK

**Keywords:** health, performance, sport, talent, training

## Abstract

The impact of National Governing Body talent development programmes on injury, illness, sleep, wellbeing and stress of developing elite athletes (DEA) is poorly understood. Therefore, we examined differences between age‐matched DEA (*n* = 42, 25 females; *M*age = 21.0; SD = 2.5) and recreationally active athletes (RAA, *n* = 79, 56 females; *M*age = 21.2; SD = 2.8) on these variables over 14 weeks of training using a weekly online monitoring tool. Compared to RAA, DEA completed a greater proportion of planned training and competition without health problems or reducing training volume. Despite training more hours (DEA *M* = 17.1; SD = 5.1, RAA *M* = 6.0; SD = 3.2, *p <* 0.001), DEA reported similar recovery, higher readiness to train, more sleep, better sleep quality, higher wellbeing (DEA *M* = 68%; SD = 15, RAA *M* = 56% SD = 16, *p <* 0.001), lower stress and fewer injuries, resulting in fewer days lost to injuries than RAA (DEA *M* = 0.4; SD = 1.5, RAA *M* = 2.5 SD = 6.7, *p =* 0.01). There was no difference between DEA and RAA in the prevalence of illness or days lost due to illness. In conclusion, despite a greater training and competition load, DEA reported better health and wellbeing than RAA, suggesting the increased demands of National Governing Body talent development programmes may not adversely affect health. These findings also highlight the benefits and importance of talent development systems undertaking a holistic and multidisciplinary approach to athlete monitoring.

## Highlights

- Little is known about the impact of training and competition demands on developing elite athletes (DEA; those athletes training and competing as part of National Governing Body talent development programs).
- DEA train and compete more than age‐matched recreationally active athletes but report better sleep, higher wellbeing, lower stress and fewer injuries, resulting in fewer training and competition days lost to injuries.
- These study findings highlight the benefits of comprehensive and multidisciplinary approaches to athlete monitoring, particularly in the DEA population.

## 1 Introduction

Achieving the highest levels of performance takes considerable time, dedication and sacrifice, with most elite athletes spending numerous hours in talent development programmes to reach the pinnacle of their sport (Bergeron et al. 2015; Güllich and Emrich 2014). Given the importance of high‐quality training in facilitating achievement, it is unsurprising that substantial research has explored the relative influence of training and its associated correlates and consequences, including injury, illness and other indices of health and wellbeing, in the context of elite performance (Drew et al. 2017; Rice et al. 2019; Walsh and Oliver 2016). In contrast, much less is known about these factors concerning developing elite athletes (DEA) in National Governing Body (NGB) talent development programmes (Rongen et al. 2018). Whilst it is evident that DEA spend considerable time training and competing, the consequences of training and competition demands are poorly understood. For example, whether DEA suffer with more injuries and illness and lose more training and competition days, due to higher training loads than similar age recreationally active athletes (RAA) is unclear. Moreover, recent literature has voiced concerns surrounding the mental health challenges of elite sport environments (Lebrun and Collins 2017; Purcell et al. 2020) and therefore, DEA may be more prone to negative impacts on health and wellbeing that stem from their training environment (Purcell et al. 2020). Alternatively, the dedicated support network available to DEA may help them to have better health and wellbeing than age‐matched RAA (Purcell et al. 2019; Rongen et al. 2018).

With these issues in mind, in the present study we explored training and competition demands, stress, illness, injury and well‐being in DEA and age‐matched RAA to quantify potential differences between these groups. Understanding whether age‐matched RAA and DEA differ in these variables is important so athletes and coaches can develop talent pathway stage‐appropriate strategies to target specific factors to improve athletes' development and avoid potential harm.

Our overarching hypothesis was, in comparison with RAA, DEA would report greater training and competition demand, that is, greater training and competition hours, poorer sleep, lower wellbeing and greater psychological stress. We further expected differences between DEA and RAA for illness and injury but refrained from making directional hypotheses. We expand on the rationale for these hypotheses below, although it should be noted that these hypotheses are based upon the available literature, which largely focuses on established elite athletes on performance programmes with limited exploration of training demands between developing elite and age‐matched recreationally active individuals.

First, it is well known that athletes complete considerable training (Güllich and Emrich 2014; Schwellnus et al. 2016; Soligard et al. 2016), so greater training and competition hours were expected in DEA than in RAA. Second, given the growing evidence that athletes obtain insufficient sleep quantity due to early morning/late evening training and competition (Leeder et al. 2012; Sargent et al. 2014; Sargent and Roach 2016; Walsh et al. 2020), we expected DEA to report shorter and poorer quality sleep than RAA. Third, as sleep influences mood and wellbeing (Consensus Conference Panel et al., 2015; Purcell et al. 2019), we hypothesised that DEA would report lower wellbeing. Fourth, although DEA receive additional support, we expected DEA to report greater stress than RAA due to the pathway‐training environment demands and the associated organisational stressors (Woodman and Hardy 2001). Fifth, in terms of injury and illness, we refrained from making directional hypotheses, as although evidence indicates that regular, prolonged and intense exercise and poor sleep, are associated with an increased illness and injury (Gao et al. 2019; Valtonen et al. 2021; Walsh et al. 2022), there is an increasing number of studies that suggest *high achieving* athletes tolerate high training demands without increased injury or illness (Mårtensson et al. 2014; Schwellnus et al. 2016; Soligard et al. 2016; Walsh and Oliver 2016).

## 2 Method

### 2.1 Participants

Forty‐two DEA (17 males, 25 (60%) females; *M*_age_ = 21.0; SD = 2.5) were recruited from four Olympic NGBs: British Rowing (*n* = 13), Swim England (*n* = 16), British Canoe Slalom (*n* = 8) and British Canoe Sprint (*n* = 5). All DEA were part of a funded national governing body talent development programme. We also recruited 79 age‐matched recreationally active athletes (RAA; 23 males, 56 (71%) females; *M*_*age*_ = 21.2; SD = 2.8). RAA participated in a variety of physical activities and sports: (badminton *n* = 4, basketball *n* = 3, boxing *n* = 1, cheerleading *n* = 2, climbing *n* = 3, dance *n* = 1, Gaelic football *n* = 1, gymnastics *n* = 2, hockey *n* = 18, horse riding *n* = 1, lacrosse *n* = 1, martial arts *n* = 6, netball *n* = 5, pole fitness *n* = 2, rowing *n* = 2, rugby *n* = 3, running *n* = 4, soccer *n* = 1, squash *n* = 2, swimming *n* = 3, trampolining *n* = 1, triathlon *n* = 1, volleyball n = 1, weightlifting/gym *n* = 11). RAA participated in these activities recreationally and completed some competitions; however, they were not sport‐contracted, semi‐professional or professional athletes. The study received institutional ethics approval (P10‐18/19) with all DEA and RAA providing written informed consent.

### 2.2 Measures

#### 2.2.1 The Athlete Monitoring Questionnaire

To facilitate the completion of the multiple variables of interest, we incorporated existing measures of these constructs into a weekly online monitoring tool that could be completed easily by participants and named it the Athlete Monitoring Questionnaire (AMQ). We followed best practice guidelines and selected questions from validated measures based on item relevance and comprehensibility to minimise athlete burden whilst retaining psychometric integrity (Horvath and Röthlin 2018). The individual question content was identical to the original, except that we modified some questions to ensure they all had the same 1‐week time domain. The constructs and associated measures were as follows: training and competition hours, session RPE (Foster et al. 2001), readiness to train, perceived recovery (Laurent et al. 2011), injury and illness prevalence and relative impact on training and performance (Clarsen et al. 2013), perceived stress (S. Cohen et al. 1983), wellbeing (Topp et al. 2015) and sleep hours, quality and latency (Buysse et al. 1989; Samuels et al. 2016). In total the AMQ contained 34 questions and was designed to be completed in approximately five minutes. A copy of the AMQ can be found in the Supporting Information S1. The reading ease of the questionnaire was high (Flesch reading ease = 88, Flesch‐Kincaid Level = 3).

### 2.3 Procedures

Participants completed the AMQ online (Qualtrics, Provo, UT, USA 2005) over a 14‐week block of regular training from October 2019 to February 2020. Each participant received a weekly individualised link to the questionnaire. Researchers supervised all participants during the first week of completion to ensure understanding and accuracy of responses. A 48‐h completion window prohibited data from one week altering responses to the next. Non‐responders received a reminder after 24‐h. Once the completion window had closed, researchers downloaded the data and generated a weekly feedback document. The RAA population could access similar feedback via a personalised login to a platform created using R.

### 2.4 Data Analysis

#### 2.4.1 Prevalence of Injury and Illness

To calculate the prevalence of all health problems, injuries and illnesses over the study period, we followed the methodology previously described by Clarsen and colleagues (Clarsen et al. 2014).

#### 2.4.2 Statistical Analyses

We analysed data using SPSS statistical software (IBMCorp. 2016. IBM SPSS Statistics for Windows, Version 24.0. Armonk, NY: IBM Corp). All participants were included in the final analyses. We averaged participant scores across the data collection period and used independent *t‐tests* to analyse between‐group differences (DEA and RAA). We calculated effect sizes using Cohen's *d*, with 95% confidence intervals, where 0.2 represented a small effect size, 0.5 a medium effect size and 0.8 a large effect size (J. Cohen 1988).

## 3 Results

### 3.1 AMQ Utility: Completion Rate and Times

The mean completion rate across the data collection was 77% (SD = 28%, DEA *M* = 83%; SD = 23%, RAA *M* = 74%; SD = 31%) with a completion time of 4 min and 41 s (SD = 2 min and 20 s, DEA *M* = 5 min and 2 s; SD = 2 min and 30 s, RAA *M* = 4 min and 19 s; SD = 2 min 15 s).

### 3.2 Training Outcomes

DEA trained and competed for more hours and at a higher perceived effort than RAA (Table 1). Despite the greater training volume, DEA reported similar perceived recovery and greater readiness to train than RAA athletes.

**TABLE 1:** A comparison of DEA and RAA for training variables.

Variable	DEA (*n* = 42)	RAA (*n* = 79)	*t* (df)	*p*	Effect size
Training and competition volume (h/wk)	17.1 (5.1)	6.0 (3.2)	12.9 (59)	< 0.001^a^	2.6 [2.0, 3.2]
RPE	5.8 (1.2)	4.5 (1.5)	4.7 (119)	< 0.001^a^	0.9 [0.5, 1.3]
Perceived recovery	6.4 (1.2)	6.1 (1.8)	1.3 (114)	0.180	0.2 [−0.1, 0.6]
Perceived readiness to train	3.7 (0.7)	3.4 (0.7)	2.2 (119)	0.028^a^	0.4 [0.1, 0.8]

*Note:* Data are presented as means (standard deviations). Effect sizes are Cohen's d with 95% confidence intervals. Perceived recovery: 0 = very poorly recovered, 10 = very well recovered; Perceived readiness to train: 0 = at no time, 5 = all the time.

Abbreviations: RPE, ratings of perceived exertion, where 0 = at rest, 10 = maximal.

^a^ Significant difference between DEA and RAA (*p* < 0.05).

### 3.3 Injury and Illness Prevalence and Severity

On average the prevalence of health problems was lower for DEA than RAA (Table 2). This difference appears attributable to fewer injuries and days lost due to injury in the DEA group rather than fewer illnesses, as DEA did not differ to RAA on illness prevalence or days lost due to illness.

**TABLE 2:** A comparison of DEA and RAA health problems.

Variable	DEA (*n* = 42)	RAA (*n* = 79)	*t* (df)	*p*	Effect size
Prevalence of all health problems (%)	17 (12)	24 (22)	−2.2 (119)	0.028^a^	−0.4 [−0.8, −0.0]
Injuries (%)	4 (7)	13 (20)	−3.5 (106)	0.001^a^	−0.6 [−1.0, −0.2]
Illnesses (%)	13 (11)	11 (15)	0.7 (119)	0.139	0.2 [−0.2, 0.5]
Average days lost per athlete due to health problems	4 (5)	6 (9)	−1.5 (119)	0.150	−0.3 [−0.6, 0.1]
Injuries	0.4 (1.5)	2.5 (6.7)	−2.6 (119)	0.010^a^	−0.4 [−0.8, −0.1]
Illnesses	4 (4)	3 (6)	0.3 (119)	0.784	0.1 [−0.3, 0.4]
Symptoms					
No symptoms (%)	73 (21)	58 (32)	3.0 (114)	0.004^a^	0.5 [0.2, 0.9]
Mild symptoms (%)	17 (13)	20 (20)	−1.0 (114)	0.279	−0.2 [−0.6, 0.2]
Substantial symptoms (%)	10 (12)	21 (27)	−3.2 (117)	0.002^a^	−0.6 [−0.9, −0.2]

*Note:* Data are presented as means (standard deviations). Effect sizes are Cohen's d with 95% confidence intervals.

Abbreviations: Severity = the cumulative severity score for each week the health problem is recorded/the number of weeks the health problem lasts.

^a^ Significant difference between DEA and RAA (*p* < 0.05).

### 3.4 Planned Training and Performance Proportion Completed

DEA were more available to participate fully without health problems in weekly training and competition than RAA (Table 3). Furthermore, DEA were more available to complete training and competition without a reduction in volume or performance.

**TABLE 3:** A comparison of DEA and RAA availability to train and compete.

Variable	DEA (*n* = 42)	RAA (*n* = 79)	*t* (df)	*p*	Effect size
Participation					
Full participation in training/competition without health problems (%)	74 (22)	57 (33)	3.3 (115)	0.001^a^	0.6 [0.2, 1.0]
Full participation in training/competition with health problems (%)	11 (16)	23 (27)	−3.2 (118)	0.002^a^	−0.6 [−1.0, −0.2]
Reduced/cannot participate in training/competition (%)	16 (12)	20 (24)	−1.1 (119)	0.291	−0.2 [−0.6, 0.2]
Training volume					
No training volume reduction (%)	80 (16)	67 (28)	3.1 (118)	0.003^a^	0.5 [0.2, 0.9]
Mild training volume reduction (%)	11 (10)	16 (17)	−1.8 (117)	0.071	−0.3 [−0.7, 0.1]
Substantial training volume reduction (%)	9 (9)	17 (23)	−2.7 (114)	0.008^a^	−0.5 [−0.8, 0.1]
Sports performance					
No sports performance reduction (%)	76 (22)	62 (32)	2.7 (110)	0.009^a^	0.5 [0.1, 0.9]
Mild sports performance reduction (%)	15 (15)	19 (19)	−1.1 (119)	0.280	−0.2 [−0.6, 0.2]
Substantial performance reduction (%)	10 (12)	19 (25)	−2.9 (118)	0.005^a^	−0.5 [−0.9, −0.1]

*Note:* Data are presented as means (standard deviations). Effect sizes are Cohen's d with 95% confidence intervals.

Abbreviations: Severity = the cumulative severity score for each week the health problem is recorded/the number of weeks the health problem lasts.

^a^ Significant difference between DEA and RAA (*p* < 0.05).

### 3.5 Sleep, Stress and Wellbeing

DEA went to bed and woke up earlier, napped more and obtained a greater quantity of sleep in 24 h than RAA (Table 4). DEA reported better sleep quality than RAA. However, both DEA and RAA reported that they experienced sleep latency and sleep disturbance on more nights than they did not. DEA reported better wellbeing and lower stress than RAA.

**TABLE 4:** A comparison of DEA to RAA for sleep, stress and wellbeing.

Variable	DEA (*n* = 42)	RAA (*n* = 79)	*t* (df)	*p*	Effect size
Total daily sleep (hh: mm)	07:51 (00:44)	07:25 (01:02)	2.7 (109)	0.008^a^	0.5 [0.1, 0.9]
Night sleep (hh: mm)	07:31 (00:50)	07:12 (01:00)	1.8 (119)	0.073	0.4 [0.0, 0.7]
Waketime (hh: mm)	06:18 (00:58)	08:57 (01:15)	−11.9 (119)	< 0.001^a^	−2.3 [‐2.8, −1.8]
Bedtime (hh: mm)	22:09 (00:38)	24:00 (01:30)	−9.4 (115)	< 0.001^a^	−1.6 [‐2.0, −1.2]
Napping duration (hh: mm)	01:04 (00:35)	00:50 (00:35)	2.0 (119)	0.045^a^	0.4 [0.0, 0.8]
Napping days per week	2 (1)	1 (1)	2.6 (119)	0.010^a^	0.5 [0.1, 0.9]
Sleep quality (% fairly/very‐bad sleep)	13 (20)	27 (30)	−3.1 (113)	0.003^a^	−0.6 [−0.9, −0.2]
Sleep latency (%)	53 (36)	69 (32)	−2.5 (119)	0.014^a^	−0.5 [−0.9, −0.1]
Sleep disturbance (%)	68 (34)	69 (33)	−0.1 (119)	0.950	0.0 [−0.4, 0.4]
Average perceived stress score (out of 16)	4.6 (2.6)	6.3 (2.5)	−3.4 (118)	< 0.001^a^	−0.7 [−1.0, −0.3]
Average wellbeing score (%)	68 (15)	56 (16)	4.2 (119)	< 0.001^a^	0.8 [0.4, 1.2]

*Note:* Data are presented as means (standard deviations). Effect sizes are Cohen's d with 95% confidence intervals.

Abbreviations: Sleep latency (%), percentage of weeks where athletes reported that on one night or more per week they were unable to sleep within 30 min; Sleep disturbance (%), percentage of weeks where athletes reported that on one night or more per week they woke up in the night; Total daily sleep, average total sleep reported within 24 h—including napping duration.

^a^ Significant difference between DEA and RAA (*p* < 0.05).

## 4 Discussion

As expected, DEA completed more training and competition hours and exerted greater effort in training. However, DEA completed a higher proportion of their planned training and competition than RAA, possibly because they reported fewer injuries. DEA also reported greater perceived readiness to train, improved wellbeing and lower stress than RAA, which may have contributed to their greater availability to train and compete.

### 4.1 Injury and Illness

DEA completed more training and competition hours without health problems than RAA and reported similar levels of health problems to Olympic athletes (Clarsen et al. 2014). The differences in training and competition observed here appear to be a function of injury as opposed to illness as DEA reported fewer injuries and days lost to injury than RAA and less variability in days lost, yet both groups reported similar levels of illness. The 4% injury prevalence in DEA was consistent with literature from elite junior athletes (Pluim et al. 2016), yet considerably lower than in professional and Olympic athletes, where studies have reported weekly injury prevalence of between 36% and 40% (Clarsen et al. 2014; Nordstrøm et al. 2020). The lower injury prevalence and lower variability in days lost to injury in DEA may be because as part of an NGB‐funded performance programme, DEA received support and regular education on load management, sleep hygiene and prevention of injury and illness (Bourdon et al. 2017). The increased variability of days lost to injury in RAA is likely a function of these athletes not being afforded the same levels of similar support as DEA on NGB‐funded programmes.

The groups did not differ in illness prevalence, with 3–4 days lost on average for each illness, which is similar to other active populations (Harrison et al. 2021). However, it is notable that the weekly prevalence of illness in both groups was higher than reported in previous research (Clarsen et al. 2014; Nordstrøm et al. 2020). The most common symptoms reported in our study were breathing difficulties (18%), cough (10%), sore throat (9%), blocked/running nose (9%) and headache (8%), all of which are associated with a respiratory infection (Dowling et al. 1958). Our data collection period, which involved the UK autumn and winter seasons, could explain these differences as influenza incidence exhibits seasonal fluctuations with a peak in the autumn and winter months (Moriyama et al. 2020).

### 4.2 Sleep

Our RAA sleep and wake times are consistent with large population studies where sleep and wake times peak in lateness at ∼19 years of age, before becoming earlier with advancing age (Fischer et al. 2017). The earlier wake time reported by DEA compared to RAA is most likely the consequence of the early morning swim and land‐based weight training completed by DEA, which is consistent with other studies examining sleep in trained swimmers and similar populations (Sargent et al. 2014). Despite earlier waking DEA reported greater total daily sleep and better sleep quality than RAA. The total daily sleep quantity reported by DEA is somewhat surprising, as previous literature has found elite athletes tend to curtail sleep (Leeder et al. 2012; Sargent et al. 2014). Our study highlights the significance of napping for DEA, as naps were more frequent and had a longer duration than RAA. Moreover, our data highlight that daytime napping was important to ensure adequate total daily sleep was obtained in DEA and on average, they met the total daily sleep recommendations (Hirshkowitz et al. 2015). The better sleep reported by DEA than RAA may in part explain why DEA reported fewer injuries and fewer days lost to injury than RAA (Charest and Grandner 2022).

### 4.3 Wellbeing and Stress

DEA reported higher wellbeing and lower stress than RAA. Wellbeing and stress levels in DEA were similar to the general population (Warttig et al. 2013) and higher than levels reported previously in younger athletic samples (Ohlert and Ott 2017). Taken together, these findings suggest that contrary to the view that some aspects of elite sport increase the risk of poor mental health (Lebrun and Collins 2017), in our sample at least, being part of an NGB performance pathway was not associated with poorer stress and wellbeing than that experienced in the general population and was better than experienced in age‐matched RAA. The higher wellbeing and lower stress of DEA coupled with improved sleep are likely indicative of adequate recovery and could also be explained, at least in part, by perceptions of support available within the pathway. Further, greater wellbeing has been reported to positively impact the training output of individuals (Gallo et al. 2016), thus, the higher weekly training hours and perceived effort, coupled with the greater perceived recovery and greater readiness to train in DEA compared to RAA, could be attributable to the self‐reported higher wellbeing and lower stress.

### 4.4 Implications

Collectively, the results highlight differences in training and related variables between DEA and RAA. DEA can complete high weekly training hours at a higher perceived effort but with a low prevalence of injuries. DEA demonstrated better wellbeing, lower stress, greater sleep duration, better sleep quality, greater perceived recovery and greater readiness to train than RAA. Overall, these data suggest that performance pathways, or at least those working with the athletes sampled here, are managing their DEA well and providing access to appropriate support (e.g. physiotherapy, mental health support) and as such, these athletes do not seem to be as susceptible to consequences associated with the pressures of established elite adult sport. That said, athletes, coaches and organisations are still able to learn from these data. Given the prevalence of respiratory illness across both groups, education on illness prevention and ways to maintain immune health (Walsh 2018) would be particularly worthwhile in DEA. Moreover, this study highlighted the importance of daytime napping to achieve daily sleep duration recommendations. Consequently, sports may benefit from reviewing athlete sleep and recommending daytime napping for athletes who do not meet sleep recommendations, particularly as napping has been shown to benefit exercise performance in those obtaining limited night‐time sleep (Blanchfield et al. 2018). Many of these recommendations could be presented via policy changes at the NGB level.

At a broader level, the wide variety of data differentiating our groups underscores the benefits of taking a holistic and multidisciplinary approach to athlete monitoring. Although different approaches to monitoring athlete training‐related behaviours and cognitions exist, most, such as GPS (Coutts and Duffield 2010), or session RPE (Foster et al. 2001) are unidimensional, measuring only one component of training and multidisciplinary tools are typically commercially licenced products that incur a financial cost (e.g. Smartabase and Metrifit). Our approach (using the AMQ) circumvents these limitations and appears well suited for monitoring training and related factors in athletes with short weekly completion times (around 5 minutes) and high completion rates, which compare favourably with those previously published for other athlete monitoring tools (Barboza et al. 2017; Gastin et al. 2013). Thus, with minimal effort, athletes and practitioners can obtain a multidisciplinary perspective on athlete training and health status.

### 4.5 Limitations

Despite the clear findings, some limitations are noteworthy. The cross‐sectional design, coupled with a relatively short window for data collection, means that we cannot generalise our findings to an athletic season or career. Future research using longitudinal designs would overcome this limitation. Yet, such an approach would require researchers to follow athletes throughout their sporting career (i.e., from recreation to developing elite, to elite) and may take many years. There would also be a benefit in sampling DEA from a wider range of sports to enhance the generalisability of the findings. Furthermore, matching DEAs and RAAs for sport type would remove the possibility of sport differences influencing findings.

Due to previous monitoring tool issues regarding utility, adherence and comprehensiveness, we used shortened validated measures reflecting a week‐long period in the AMQ. Although we engaged in a rigorous process of measure and item selection, some of the measures may be more susceptible to recall bias across a week, as opposed to more immediate ratings, that is, daily. While we acknowledge daily ratings benefit recall, they increase athlete burden and can lead to substantially reduced adherence rates, negating the benefit of daily monitoring. Additionally, whilst our findings provide some evidence of validity in the sampled populations and sports, future research should extend this work to more populations and sports.

We believe that revisions to our monitoring approach would be worthwhile. For example, we did not differentiate between training and rest day sleep, nor did we consider potential differences between weekdays and weekends. Such additions may be of interest to practitioners, considering recent evidence suggesting performance may be enhanced with ‘sleep banking’ (Arnal et al. 2016; Vitale et al. 2019), that is, deliberate planning of more sleep when anticipating future sleep loss. Second, collecting details regarding the intended and actual days of training per week and the reason for any discrepancy would add accuracy to the implications of days missed due to health problems. Thirdly, combining activity monitors with our weekly questionnaire data would have complemented and strengthened the self‐reported sleep and training hour data and given that wearable activity monitors are increasingly common, may be an approach to pursue in future research studies and by athletes in practice.

## 5 Conclusions

In comparison with age‐matched RAA, DEA completed greater training and competition load and reported better recovery, including more sleep, lower psychological stress, better wellbeing, fewer injuries and days lost to injury. These findings indicate the NGB talent development pathways sampled here manage their athletes' training, wellbeing and health well. Further, these findings suggest that greater training and competition load, better sleep and wellbeing and lower injury prevalence and stress are associated with being a developing elite athlete. As such, researchers and applied practitioners working in these environments may benefit from regular monitoring of these important outcomes.

## Funding

This research was part of the Pathway2Podium project, funded by UK Sport, Bangor University and the Economic and Social Research Council (via the ESRC Wales Doctoral Training Partnership). The project was delivered in conjunction with the UK Sports Institute (formerly the English Institute of Sport) and National Governing Bodies. The research aimed to enhance UK Sport's talent development pathway. The Pathway2Podium project was led by Dr. Gavin Lawrence, Prof. Tim Woodman and Dr. Ben Holliss.

## Ethics Statement

The study received institutional ethical approval (no: P10‐18/19).

## Consent

All participants provided written consent.

## Conflicts of Interest

The authors declare no conflicts of interest.

## Supporting information


Supporting Information S1
