# Supplementary material for: A Comparison of Training, Injury, Illness, Sleep, Wellbeing and Stress Between Developing Elite and Recreational Athletes
Source: Eur J Sport Sci. 2025 Nov 23;25(12):e70093. doi: 10.1002/ejsc.70093 (PMC12640734; doi:10.1002/ejsc.70093)
Supplement: Supplementary file 1 — Supporting Information S1 [file EJSC-25-e70093-s001.docx]

##

## Supplementary information

## Athlete Monitoring Questionnaire (AMQ)

You are about to complete a monitoring tool that asks about your training, recovery, sleep, overall well-being and any injury, illness or other health problems experienced over the past week (*depending on completion day detail the day from and to e.g., Monday to Sunday).* Please try to answer the questions as thoroughly and as honestly as possible. There are no right or wrong answers. Your answers will not affect your position on the programme.

TRAINING:

Please take a few seconds to think back over the last week of training. Think about where you spent most of the time training, whom you trained with, and the most memorable session of the week. It may help you to have your training diary with you to aid in completion.

Question 1:

How many hours of the following have you completed in the past week?

- Hours of sport-specific:
- Hours of other training (e.g., gym, weights, yoga, rehabilitation etc.):
- Hours of competition:

Question 2:

Please briefly describe the types of training that you have been doing in the past week.

________________________________________________________________

________________________________________________________________

________________________________________________________________

Question 3: (If relevant)

How many kilometres (km) have you *covered* in the past week?

Question 4:

How many days have you competed on this past week?

Question 5:

How many times have you raced/competed?

Question 6:

Over the past week, how often have you felt ready to train?

- All the time (5)
- Most of the time (4)
- More than half the time (3)
- Less than half the time (2)
- Some of the time (1)
- At no time (0)
- I have not trained (6)

Question 7:

Over the past week, how often have you felt motivated to train?

- All the time (5)
- Most of the time (4)
- More than half the time (3)
- Less than half the time (2)
- Some of the time (1)
- At no time (0)
- I have not trained (6)

Question 8:

Over the past week, how well recovered have you felt?

- 0 Very poorly recovered (0)
- 1 (1)
- 2 Not well recovered (2)
- 3 (3)
- 4 Somewhat recovered (4)
- 5 Adequately Recovered (5)
- 6 Moderately Recovered (6)
- 7 (7)
- 8 Well Recovered (8)
- 9 (9)
- 10 Very well recovered (10)
- I have not trained (11)

Question 9:

Over the past week, what has been your overall perceived exertion?

- 0 At rest (0)
- 1 Very, very easy (1)
- 2 Easy (2)
- 3 Moderate (3)
- 4 Somewhat Hard (4)
- 5 Hard (5)
- 6 (6)
- 7 Very Hard (7)
- 8 (8)
- 9 (9)
- 10 Maximal (10)
- I have not trained (11)

HEALTH:
Please answer all questions regardless of whether you have experienced health problems in the past week. Select the alternative that is most appropriate for you, and in the case that you are unsure, try to answer as best you can anyway.
A health problem is any condition that you consider to be a reduction in your normal state of full health, irrespective of its consequences on your sports participation or performance, or whether you have sought medical attention. This may include but is not limited to, injury, illness, pain, or mental health conditions. If you have several health problems, please begin by recording your worst problem in the past 7 days. You will have a chance to register other problems at the end of the questionnaire.

Question 10a: Participation
Have you had any difficulties participating in normal training and competition due to injury, illness, or other health problems during the past week?

- Full participation without health problems (injury/illness) (0)
- Full participation, but with injury/illness (8)
- Reduced participation due to injury/illness (17)
- Cannot participate due to injury/illness (25)

Q10b: Modified training/competition
To what extent have you reduced your training volume due to injury, illness, or other health problems during the past week?

- No reduction (0)
- To a minor extent (6)
- To a moderate extent (13)
- To a major extent (19)
- Cannot participate at all (25)

Q10c: Performance
To what extent has injury, illness or other health problems affected your performance in training during the past week?

- No effect (0)
- To a minor extent (6)
- To a moderate extent (13)
- To a major extent (19)
- Cannot participate at all (25)

Q10d: Symptoms
To what extent have you experienced symptoms/health complaints during the past week?

- No symptoms/health complaints (0)
- To a mild extent (8)
- To a moderate extent (17)
- To a severe extent (25)

**Skip Logic: If Q10a, Q10b, Q10c and Q10d = 0 then skip to Question 17**

If you have several illnesses or injury problems, please refer to the one that has been your worst problem this week.

Question 11:
Is the health problem referred to in the four questions above an injury or an illness?

- Injury
- Illness
- No injury, illness, or health problem

**Skip Logic:**

**If Q11 = Injury continues to Question 12a**

**If Q11 = Illness, then skip to Question 13a**

**If Q11 = No injury, illness or health problem then skip to Question 17**

INJURY

Question 12a:

Please select the box that best describes the location of your injury. If the injury involves several locations, please select the main areas. If you have multiple injuries, you will have a chance to register other problems at the end of the questionnaire.


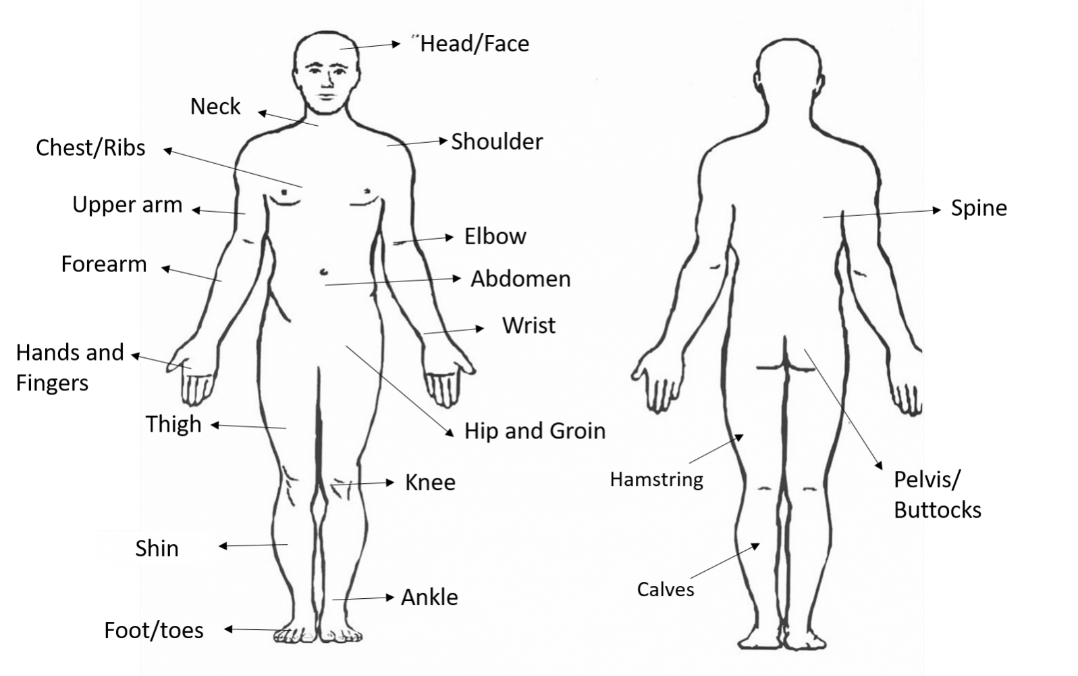


Question 12b:

Please indicate whether the injury location is on the right or left of your body.

- Right (1)
- Left (2)
- Both Sides (4)
- Other, please specify: ________________________________________________

Question 12c:

If you are aware of the type/nature of the injury you have sustained, please indicate below (if you are unsure, please tick 'I don't know).

- Concussion (regardless of loss of consciousness)
- Fracture (traumatic)
- Stress fracture (overuse)
- Other bone injuries
- Dislocation
- Tendon rupture
- Ligament rupture
- Sprain (injury of joint and/or ligaments)
- Lesion of meniscus or cartilage
- Strain/muscle rupture/tear
- Bruise
- Laceration/abrasion/skin lesion
- Dental Injury/broken tooth
- Other, please specify: ________________________________________________
- I don't know

**Now skip to Question 14**

ILLNESS

Question 13a:
Please select the boxes corresponding to the major symptoms you have experienced during the past 7 days. You may select several alternatives; however, in the case that you have several unrelated illnesses please register them at the end of the questionnaire.


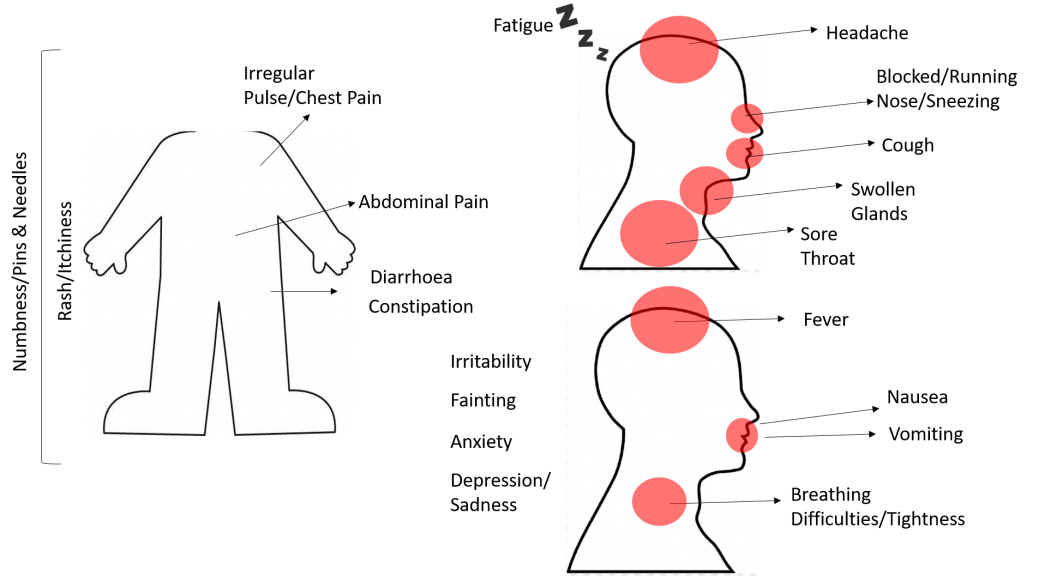


Question 13b:

If the symptoms experienced are not listed, please describe your symptoms below (including any diagnosis from medical professionals).

________________________________________________________________

________________________________________________________________

________________________________________________________________

HEALTH PROBLEM IMPACT

Question 14:
Please state the number of days over the past 7-day period that you have had to completely miss training or competition due to this problem. (You may indicate half days as well – use .5 to indicate).

Question 15:
Is this the first time you have registered this problem through this monitoring system?

- Yes, this is the first time
- No, I have reported the same problem in one of the previous two weeks
- No, I have reported the same problem previously, but it was more than two weeks ago

Question 16:
Have you experienced any other illnesses, injuries, or other health problems during the past 7 days?

- Yes, please provide a summary (e.g., injury or illness, area of the body affected, performance/training effect, including any diagnosis from medical professionals). ________________________________________________
- No other health problems to report

SLEEP

The following questions relate to your usual sleep habits during the past week only. The first set of questions asks about your usual sleep habits on days that you train, and the second set asks about your usual sleep habits on rest days.

Question 17

Q17a: On training days during the past week, what time have you usually gone to bed at night? Please report using the 24hr clock (00:00, e.g., 22:00 for 10 pm).

Q17b: On training days during the past week, what time have you usually gotten out of bed in the morning? Please report using the 24hr clock (00:00, e.g., 07:00 for 7 am).

Q17c: On rest days during the past week, what time have you usually gone to bed at night? Please report using the 24hr clock (00:00, e.g., 22:00 for 10 pm).

Q17d: On rest days during the past week, what time have you usually gotten out of bed in the morning? Please report using the 24hr clock (00:00, e.g., 07:00 for 7 am).

Question 18:

Q18a: On training days during the past week, how many hours of actual sleep did you get at night (this may be different from the number of hours you spend in bed)? Please report using the format hh:mm (e.g., 8 hours 30 minutes = 08:30).

Q18b: On rest days during the past week, how many hours of actual sleep did you get at night (this may be different from the number of hours you spend in bed)? Please report using the format hh: mm (e.g., 8 hours 30 minutes = 08:30).

Question 19:
How would you rate your sleep quality overall? Please choose one which is closest to your sleep quality over the past week.

- Very Good (1)
- Fairly Good (2)
- Fairly Bad (3)
- Very Bad (4)

Question 20:

Please indicate how often you had trouble sleeping because of you…

|  | Not during the past week (1) | Less than once a week (2) | Once or twice a week (3) | Three or more times a week (4) |
| --- | --- | --- | --- | --- |
| …cannot get to sleep within 30 minutes. (1) |  |  |  |  |
| …wake up in the middle of the night or early morning. (2) |  |  |  |  |

Question 21a

How many days over the past week have you had a nap during the daytime?

0, 1, 2, 3, 4, 5, 6, 7

Question 21b:

On the days when you napped, on average how many hours would you nap? (> = greater than, < = less than)

- Less than 0.5 hour
- > 0.5 hours but < 1 hour
- > 1 hour but < 1.5 hours
- > 1.5 but < 2 hours
- > 2 but < 2.5 hours
- > 2.5 but < 3 hours
- > 3 but < 3.5 hours
- > 3.5 but < 4 hours
- More than 4 hours

WELLBEING:

Question 22:

Please indicate for each of the ﬁve statements which is closest to how you have been feeling over the past week.

|  | At no time | Some of the time | Less than half the time | More than half the time | Most of the time | All the time |
| --- | --- | --- | --- | --- | --- | --- |
| I have felt cheerful and in good spirits |  |  |  |  |  |  |
| I have felt calm and relaxed |  |  |  |  |  |  |
| I have felt active and vigorous |  |  |  |  |  |  |
| I woke up feeling fresh and rested |  |  |  |  |  |  |
| My daily life has been ﬁlled with things that interest me |  |  |  |  |  |  |

Question 23:

The questions in this scale ask you about your feelings and thoughts during the past week. In each case, please indicate how often you felt/thought a certain way in the past week.

|  | Never | Almost Never | Sometimes | Fairly Often | Very Often |
| --- | --- | --- | --- | --- | --- |
| How often have you felt that you were unable to control the important things in your life? |  |  |  |  |  |
| How often have you felt confident about your ability to handle your problems? |  |  |  |  |  |
| How often have you felt that things were going your way? |  |  |  |  |  |
| How often have you felt difficulties were piling up so high that you could not overcome them? |  |  |  |  |  |
